# Genetics and other biomarkers of treatment resistance in depression

**DOI:** 10.1192/j.eurpsy.2025.105

**Published:** 2025-08-26

**Authors:** C. Fabbri

**Affiliations:** Department of Biomedical and NeuroMotor Sciences, University of Bologna, Bologna, Italy

## Abstract

**Abstract:**

Major depressive disorder (MDD) is the most heterogeneous psychiatric disorder, as over 1400 combinations of symptoms can satisfy DSM diagnostic criteria, and understanding the underlying biological heterogeneity is fundamental in the perspective of precision psychiatry. Genetic variants are responsible for part of this heterogeneity and contribute to inter-individual differences in treatment effects. Therefore, pharmacogenetic studies can identify predictors of poor treatment response and treatment-resistant depression (TRD), aid the prescription and development of targeted treatments or the prescription of early intensive treatments in patients at risk of TRD. Previous genome-wide association studies (GWASs) had limited power to identify specific variants/genes involved in TRD, but polygenic overlap between poor antidepressant response/TRD and several other traits was demonstrated, e.g., schizophrenia, attention deficit hyperactivity disorder (ADHD), and cognitive traits. As suggested by the high clinical heterogeneity of MDD, considering specific symptom profiles can improve the power to identify genetic signals associated with TRD. Other promising strategies to identify the biological mechanisms involved in TRD include the study of large population cohorts, using proxies of TRD defined from electronic health records (EHRs), and the integration of biomarkers reflecting also environmental factors, such as proteomics. These strategies and ongoing studies will be discussed during this talk, with reference to Psych-STRATA, a project within the Horizon Europe research and innovation programme (101057454; https://psych-strata.eu).

**Disclosure of Interest:**

None Declared

